# Isolation and identification of a feather degrading *Bacillus tropicus* strain Gxun-17 from marine environment and its enzyme characteristics

**DOI:** 10.1186/s12896-022-00742-w

**Published:** 2022-03-20

**Authors:** Naikun Shen, Mengying Yang, Chenjie Xie, Jiangxin Pan, Kunrong Pang, Hongyan Zhang, Yibing Wang, Mingguo Jiang

**Affiliations:** Guangxi Key Laboratory for Polysaccharide Materials and Modifications, Guangxi Key Laboratory of Microbial Plant Resources and Utilization, School of Marine Sciences and Biotechnology, Guangxi University for Nationalities, Nanning, 530006 China

**Keywords:** Keratinase, *Bacillus tropicus*, Fermentation conditions, Biochemical characterisation, Feather degradation mechanism

## Abstract

**Background:**

Feathers are the most abundant agricultural waste produced by poultry farms. The accumulation of a large number of feathers not only seriously pollutes the environment but also causes the waste of protein resources. The degradation of feather waste by keratinase-producing strains is currently a promising method. Therefore, screening high-producing keratinase strains from marine environment and studying the fermentation conditions, enzymatic properties and feather degradation mechanism are crucial for efficient degradation of feathers.

**Results:**

A novel efficient feather-degrading bacteria, Gxun-17, isolated from the soil sample of a marine duck farm of Beibu Gulf in Guangxi, China, was identified as *Bacillus tropicus*. The optimum fermentation conditions were obtained by single factor and orthogonal tests as follows: feather concentration of 15 g/L, maltose concentration of 10.0 g/L, MgSO_4_ concentration of 0.1 g/L, initial pH of 7.0 and temperature of 32.5 °C. The strain completely degraded the feathers within 48 h, and the highest keratinase activity was 112.57 U/mL, which was 3.18-fold that obtained with the basic medium (35.37 U/mL). Detecting the keratinase activity and the content of sulphur-containing compounds in the fermentation products showed that the degradation of feathers by the strain might be a synergistic effect of the enzyme and sulphite. The keratinase showed optimal enzyme activity at pH 7.0 and temperature of 60 °C. The keratinase had the best performance on the casein substrate. When casein was used as the substrate, the K_m_ and V_max_ values were 15.24 mg/mL and 0.01 mg/(mL·min), respectively. Mg^2+^, Ca^2+^, K^+^, Co^2+^, Al^3+^, phenylmethylsulphonyl fluoride and isopropanol inhibited keratinase activity, which indicated that it was a serine keratinase. Conversely, the keratinase activity strongly increased with the addition of Mn^2+^ and β-mercaptoethanol.

**Conclusions:**

A novel feather-degrading *B. tropicus* Gxun-17 was obtained from marine environment. The strain adapted the extreme conditions such as low temperature, high salt and high pressure. Thus, the keratinase had high activity, wide range of temperature and pH, salt tolerance and other characteristics, which had potential application value.

**Supplementary Information:**

The online version contains supplementary material available at 10.1186/s12896-022-00742-w.

## Background

With increasing commercial poultry processing, feather waste is being dumped in large amount as a by-product. Feathers contain over 90% of keratin, a kind of hard fibrous protein, which mainly exists in hair, feathers, scales, hooves, nose, horns, claws and other structures [1]. Owing to a large number of disulphide, hydrogen and hydrophobic bonds, keratin is not degraded by commonly known proteases like trypsin, pepsin and papain [2]. Physical and chemical methods of feather treatment resulted in the great loss of essential amino acids and the serious pollution to air, soil and water. Using keratinase-producing strains to degrade feathers is a promising and utilised method at present. The method has slight effect on the environment, and converts a solid contaminant into nutritionally protein-rich feedstuff for livestock [3].

Diverse groups of strains such as fungi, actinomycete and bacteria are reported to produce keratinase. Fungi are rarely used in applications because their pathogenicity and keratinase from actinomycete is far from being fully explored [4, 5]. Amongst bacteria, *Bacillus cereus*, *Bacillus subtilis, Bacillus licheniformis* and other *Bacillus* sp. are beneficial to degrade feather because of their safety and fast growth rate [6–8]. Owing to the significant difference in keratin-degrading strains, the keratinase produced by microorganisms differs significantly. The optimisation process, either through single factor, orthogonal and response surface tests, has been used to enhance keratinase activity given that keratinase production is grossly influenced by fermentation conditions and medium compositions. The construction of optimal fermentation conditions improved keratinase activity of *B. licheniformis* ALW1 by 2.9-fold (72.2 U/mL) [8]. Similarly, Mohamad et al. reported an enhanced keratinase activity from a feather-degrading *Pseudomonas* sp. LM19 after the optimisation of conditions [9]. After optimisation, keratinase activity of *Stenotrophomonas maltophilia* DHHJ reached 10.0 U/mL [10].

Keratinase (E.C. 3.4.99.11) is a class of proteases with keratinising activities. The identified keratinases are generally classified as serine or metallo protease family; for example, *B. zhangzhouensis* is a serine protease and *Chryseobacterium* sp. kr6 is a metallo protease [11, 12]. Studies have shown that keratinases from different sources show different characteristics such as optimal pH, temperature, tolerances to metal ions and chemical agents. Keratinase exhibits the enzyme activity at temperatures ranging from 30 to 70 °C or higher, and the optimal temperature of *Fervidobacterium islandicum* AW-1 is 100 °C [13–15]. The optimum pH for most keratinases is between 7.0 and 10.0, whilst the optimal pH for *Trichophyton schoenleinii* is 5.5 [16–18]. Moridshahi et al. reported that the effect of metal ions on keratinase from *B. zhangzhouensis*, Ca^2+^, K^+^, Na^+^ and Mn^2+^ could improve enzyme activity [11]. As reported by Akram et al., keratinase activity from *Bacillus* sp. NKSP-7 was promoted by mercaptoethanol, whilst enzyme activity was completely inhibited by phenylmethylsulphonyl fluoride (PMSF) [19]. In addition, keratinase can be used in several fields such as biomedicine, detergents, animal feed, leather production and wastewater treatment.

Although bacterial keratinase shows a potential to be utilised for feather bioconversion, enhancement of enzyme activities and increase in yields are required to make keratinases suitable for industrial applications. In addition, the degradation mechanism of feather keratin remains unknown, and isolating different microorganisms is important. The recognised microbial degradation process involves three essential steps: denaturation, decomposition and transamination. The existing theories of microbial degradation of feathers mainly include biological membrane potential theory, mechanical pressure theory, enzymolysis theory and thiolysis theory, the core of which is the fracture of disulphide bond [20]. Disulphide bond rupture has been found in recent years to be related to not only disulphide bond reductase but also some chemical reductants. Yamamura et al. isolated disulphide bond reductase from fermentation broth of *S. maltophilia* and confirmed that disulphide bond reductase could promote feather degradation [21]. Onifade et al. found that sulphite could degrade feathers [22].

In this study, a high-yielding keratinase strain was screened from the soil sample of a marine duck farm of Beibu Gulf in Guangxi, China, and the fermentation conditions and enzymatic properties of strain were studied. At the same time, the feather degradation mechanism by microorganisms was preliminarily explored by detecting the enzymes and sulphur-containing compounds in the fermentation broth, which provided theoretical guidance for improving the biodegradation of keratin.

## Results

### Isolation and screening of keratinase-producing strains

**Fig. 1:**
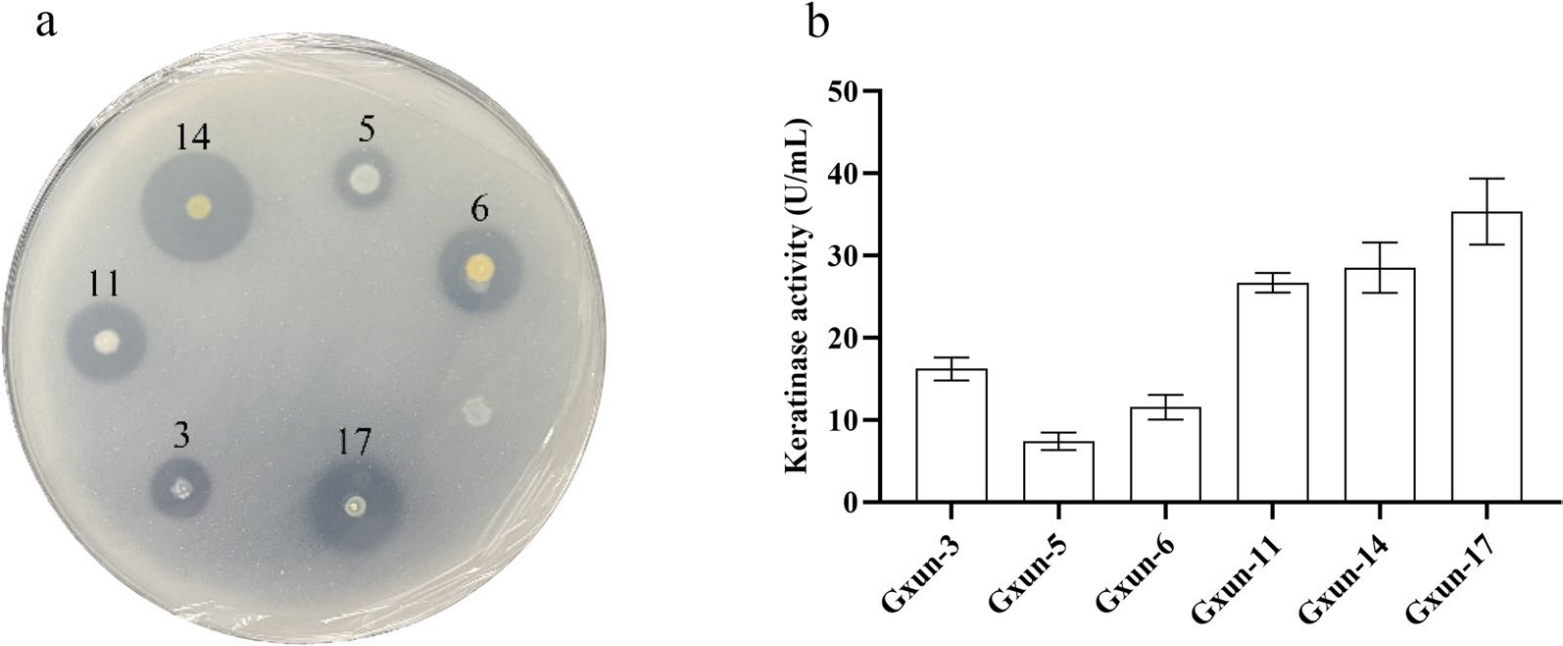
Isolation and screening of keratinase-producing strains: **a** Zone of hydrolysis around the bacterial growth on the casein plate; **b** comparison of keratinase activity by various bacteria strains

Fifteen microorganisms were obtained when the feather soil was inoculated on casein plates at 30 °C for 48 h. Then, six bacteria isolates obtained showed clear zone of hydrolysis. Keratinase assay for isolated strains was performed. Strains Gxun-11, Gxun-14 and Gxun-17 showed high feather degradation abilities and proteolytic activities, as shown in Fig. 1. The keratinase activity was highest in strain Gxun-17 of 35.37 U/mL and clear zone of hydrolysis of 13 mm. Strains Gxun-14 and Gxun-11 followed with keratinase activity of 28.56 and 26.70 U/mL and hydrolysis zones of 15 and 12 mm.

### Identification of strain Gxun-17

**Fig. 2:**
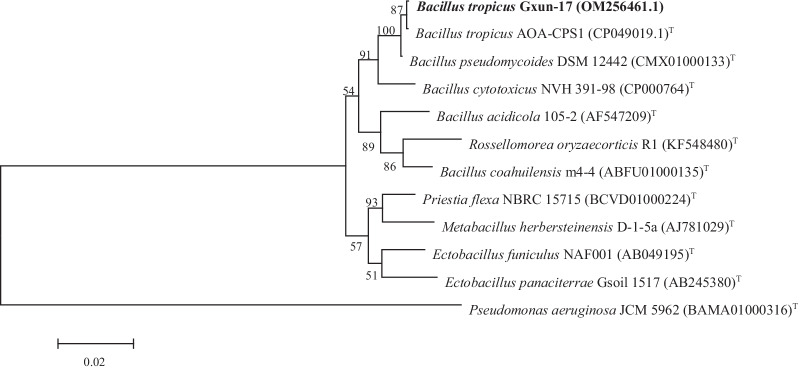
Phylogenetic tree of the isolated Gxun-17 with reference type strains based on 16S rRNA gene sequence alignment. Bootstrap values were based on 1000 replicates. Numbers in parentheses are accession numbers of published sequences in GenBank

The strain Gxun-17 from the above mentioned data was identified, and its general characteristics are stated in Table 1. Strain Gxun-17 was oxidase positive, it was negative to indole production and it could hydrolyse casein and gelatine. The strain was negative for urease activity, catalase activity, arginine hydrolase activity and hydrogen sulphide production, and it was negative for Voges Proskauer and Methyl red’s test. Strain Gxun-17 was large and irregular with white colour. Microscopic observations of the strain showed single straight gram-positive rod-shaped and approximately 1.1 μm × 2.8 μm in size (Additional file 1: Fig. S1). Further identification was supported by the 16S rRNA sequencing. BLAST results showed that the coverage rate of the 16S rRNA gene sequence of isolate Gxun-17 and *Bacillus tropic*us strain AOA-CPS1 (CP049019.1) was 100%, the E-value was 0 and the identity was 99.81%. Phylogenetic relationship between isolate Gxun-17 and its high 16S rRNA sequence similarity strains was evaluated by MEGA version 5. As observed, the isolate Gxun-17 was situated in the same clade with *B. tropicus* strain AOA-CPS1 (CP049019.1) from the phylogenetic tree (Fig. 2). The 16S rRNA sequence of the isolate Gxun-17 was submitted to the GenBank with the accession number OM256461.1. Thus, the isolate Gxun-17 was identified to be a strain of *B. tropicus* and named as *B. tropicus* Gxun-17.

**Table 1:** Biochemical and physiological characteristics of the isolate Gxun-17

Test items	Results	Test items	Results
Urease	−	Voges-Proskauer	−
Oxidase	+	Methyl red	−
Indole	−	Gelatine	+
Arginine hydrolase	−	Catalase	−
Hydrogen sulphide	−	Casein	+

+ : Positive; − : Negative

## Optimisation of fermentation conditions

### Single factor test

**Fig. 3:**
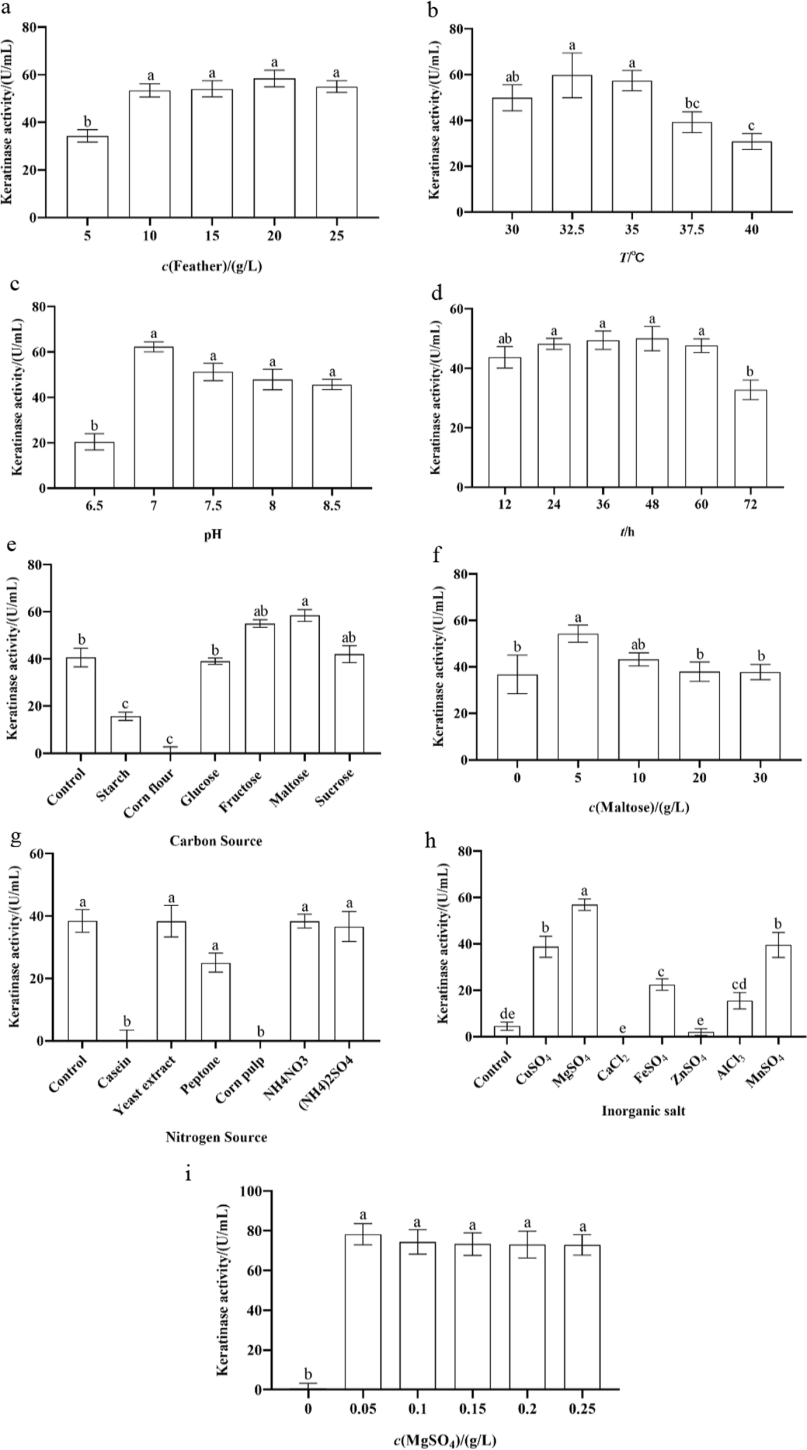
Effects of different factors on enzyme production of *B. tropicus* Gxun-17: **a** feather concentration; **b** fermentation temperature; **c** initial pH; **d** fermentation time; **e** carbon sources; **f** maltose concentration; **g** nitrogen sources; **h** inorganic salts; **i** MgSO_4_ concentration. Letters a, b, c, d and e indicate significance difference amongst the various treatments (*P* < 0.05)

The obtained results highlighted that the increase in feather concentration from 5 to 20 g/L resulted in the stepwise increase in keratinase activity from 34.34 to 58.44 U/mL. Beyond the optimum feather concentration, keratinase activity dropped and the lowest enzyme activity was 55.04 U/mL at 25 g/L (Fig. 3a). The maximum keratinase activities were 59.71 and 62.29 U/mL at the fermentation temperature and initial pH of 32.5 °C and 7.0, respectively. However, further increments in temperature and pH repressed the enzyme activity (Fig. 3b, c). Keratinase activity was low during the first 12 h, but the enzyme activity reached its peak of 50.01 U/mL as the bacteria grew at 48 h. Prolongation of fermentation time resulted in a continuous decline in enzyme activity, which reached 32.82 U/mL at 72 h (Fig. 3d). Subsequently, the fermentation medium was supplemented with various carbon sources, and the keratinase activities were inhibited in the presence of starch and corn flour compared with the control. The supplementation of the medium with glucose and sucrose did not produce any conspicuous effect. However, the enzyme activities were significantly promoted and reached 55.04 and 58.44 U/mL in the presence of fructose and maltose, respectively (Fig. 3e). The influence of maltose concentration was further assessed, and the finding indicated that 5 g/L was optimal for keratinase activity of *B. tropicus* Gxun-17 (Fig. 3f). Compared with the control, the addition of nitrogen sources including yeast extract, NH_4_NO_3_ and (NH_4_)_2_SO_4_ in the medium had no obvious effect on the keratinase activity. In the meantime, the addition of casein, corn pulp and peptone had inhibition effect. Therefore, *B. tropicus* Gxun-17 did not require nitrogen sources for fermentation (Fig. 3g). The effect of inorganic salts on enzyme activity showed that ZnSO_4_ and CaCl_2_ nearly completely inhibited the keratinase activity, whilst keratinase activity was promoted with FeSO_4_, MgSO_4_, MnSO_4_, CuSO_4_ and AlCl_3_ (Fig. 3h). The maximum stimulatory effect was obtained after MgSO_4_ supplementation, and the highest keratinase activity was 78.26 U/mL when concentration was 0.05 g/L (Fig. 3i).

### Optimum level determination using orthogonal test

The L9 (3^4^) orthogonal table was used in the experiment, and the factors and levels were set up, as shown in Table 2. The factors were selected as maltose concentration, feather concentration, initial pH and MgSO_4_ concentration, and each factor was taken at three levels.

**Table 2:** Factor and levels L9 (3^4^) of orthogonal design test

Levels	A Maltose concentration (g/L)	B Feather concentration (g/L)	C Initial pH	D MgSO_4_ concentration (g/L)
1	5	20	7.0	0.05
2	0	25	7.5	0.10
3	10	15	8.0	0.15

According to the results of orthogonal test in Table 3, the influence order of four factors was A > D > C > B. In other words, maltose concentration had the greatest influence on enzyme production of *B. tropicus* Gxun-17, followed by MgSO_4_ concentration, initial pH and feather concentration. Amongst them, the combination A_3_B_3_C_1_D_2_ was optimal and the optimum conditions were maltose concentration of 10 g/L, feather concentration of 15 g/L, initial pH 7.0 and MgSO_4_ concentration of 0.1 g/L. Three groups of validation tests were conducted because the optimum combination did not appear in the orthogonal test table, and the final keratinase activity was 112.57 ± 6.11 U/mL, which was 3.18-fold higher than the activity of basic medium.

**Table 3:** Optimization of fermentation conditions using orthogonal test

Test no	Factor				Keratinase activity (U/mL)
	A	B	C	D	
1	1	1	1	1	74.81 ± 2.69^b^
2	1	2	2	2	73.20 ± 5.56^b^
3	1	3	3	3	79.93 ± 5.31^b^
4	2	1	2	3	19.76 ± 4.84^c^
5	2	2	3	1	3.81 ± 3.64^d^
6	2	3	1	2	79.23 ± 7.38^b^
7	3	1	3	2	107.82 ± 4.63^a^
8	3	2	1	3	102.30 ± 3.50^a^
9	3	3	2	1	71.81 ± 1.15^b^
k_1_	75.98	67.46	85.45	50.14	
k_2_	34.27	59.77	54.92	86.75	
k_3_	93.98	76.99	63.85	67.33	
R	59.71	17.22	21.59	36.61	
The impact of factors	A > D > C > B				
Optimization level	A_3_	B_3_	C_1_	D_2_	

Superscript letter(s) a, b, c and d down the column were used to indicate significant difference at *P* < 0.05

### Characterisation of keratinase

**Fig. 4:**
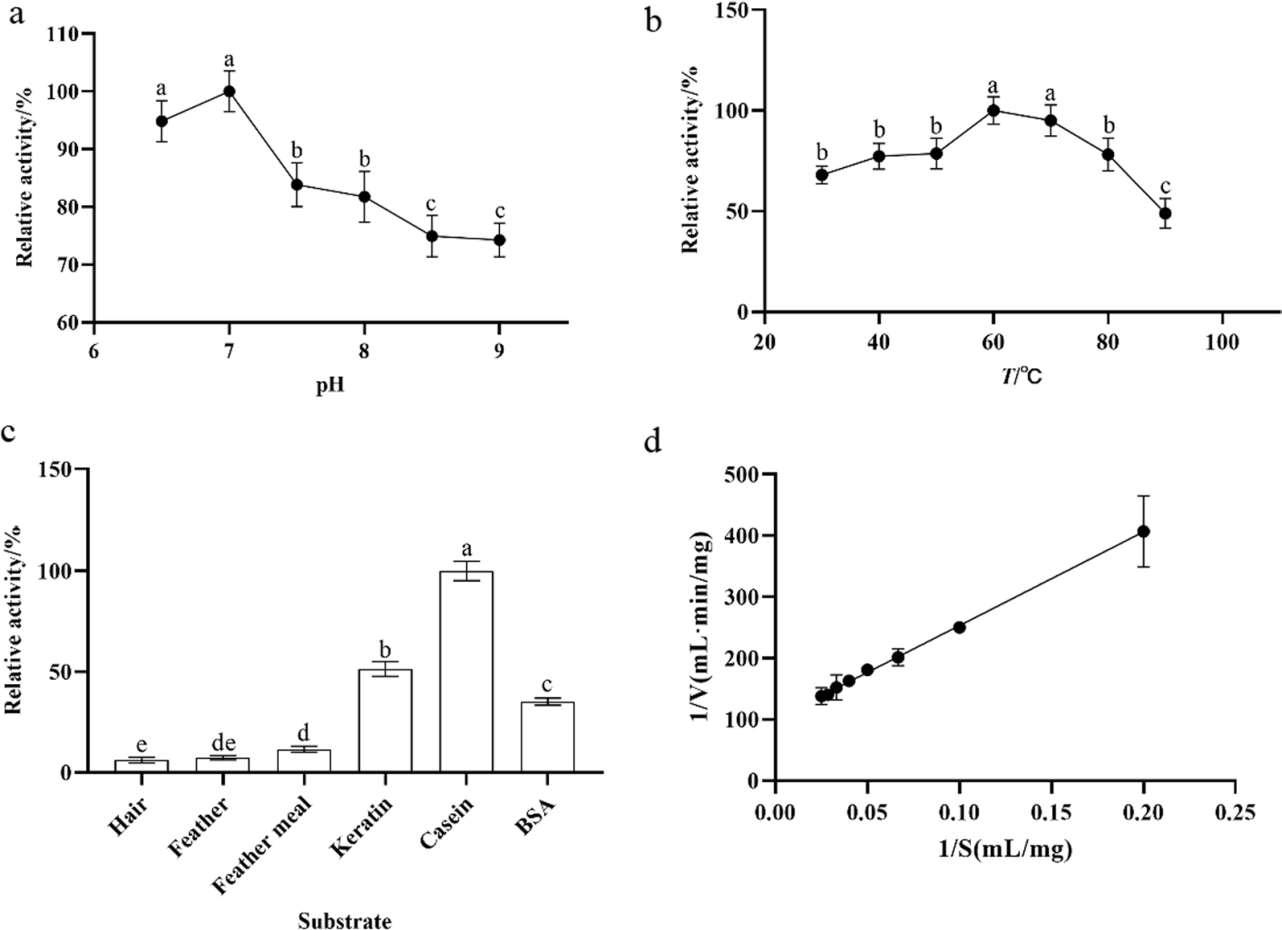
Summary of biochemical characteristics of keratinase: **a** pH optimum; **b** temperature optimum; **c** substrate specificity; **d** kinetics. Letters a, b, c, d and e indicate significance difference amongst the various treatments (*P* < 0.05)

The keratinase had higher enzyme activity from weakly acidic to weakly alkaline condition, and keratinase displayed the maximal activity at pH 7.0, which reached 89.81 U/mL. With the increase in pH, the activity decreased markedly, but it still maintained the relative activity of 70% (Fig. 4a). The activity increased following the rise in temperature with the highest activity at 60 °C. Keratinase demonstrated more than 80% relative activity at 60 °C–80 °C, and the relative activity was lowest at 90 °C of 48.96% (Fig. 4b). We found that the keratinase activity was insignificantly affected by ethylene diamine tetraacetic acid (EDTA), sodium dodecyl sulphate (SDS) and dimethyl sulphoxide (DMSO) (5%), whilst PMSF and isopropanol significantly inhibited activity (relative activity of < 40% in each case). When the keratinase was mixed with 5% isopropanol, the relative activity was 13.91%, but it increased moderately when the reagent was present at 2.5%. The keratinase activity was nearly lost under the action of PMSF at 2.5 and 5 mM. We also found that mercaptoethanol significantly enhanced enzyme activity. Relative activity increased by 395.44% in the presence of 2.5% mercaptoethanol, and the activity was also increased when the concentration rose to 5% (Table 4). The effect of metal ions on the keratinase activity showed that Na^+^, Mg^2+^, Ca^2+^, Co^2+^ and K^+^ gradually inhibited the enzyme activity with the increase in concentration from 0.025 M to 2.5 M. The relative activity was still more than 70% when the concentration of Na^+^ was 2.5 M. Al^3+^ at 0.025 M had no effect on the enzyme activity, but the enzyme activity decreased in the concentrations of 0.25 and 2.5 M. Cu^2+^ inhibited the enzyme activity at all three concentrations and was completely lost under the action of Cu^2+^ at 0.025 M. Si^2+^ decreased the enzyme activity at 2.5 M whilst increased it at 0.05 and 0.25 M. Mn^2+^ enhanced the enzyme activity at all three concentrations, and the maximum activity was observed at 0.25 M (relative activity was 260.72%) (Table 5). The keratinase showed the minimum activity when the substrate was hair (6.22 U/mL), whilst casein was the best substrate for keratinase production by *B. tropicus* Gxun-17, and the enzyme activity reached 72.02 U/mL (Fig. 4c). When casein was used as the substrate, the linear relation between 1/V and 1/S obtained by the Lineweaver–Burk double reciprocal plotting method (Y = 1529X + 100.3), and the linear relationship between R^2^ was 0.9991. The K_m_ and V_max_ values were 15.24 mg/mL and 0.01 mg/(mL·min) by calculating, respectively (Fig. 4d).

**Table 4:** Effect of various chemical agents on the keratinase

Chemical reagent	Concentration (mM)	Residual activity (%)	Chemical reagent	Concentration (%; v/v)	Residual activity (%)
None	–	100.00 ± 1.25^d^			
EDTA	2.5	98.57 ± 0.55^d^	Isopropanol	2.5	32.85 ± 2.04^e^
	5	97.15 ± 1.14^d^		5	13.91 ± 6.62^f^
SDS	2.5	98.23 ± 1.61^d^	DMSO	2.5	115.00 ± 4.55^c^
	5	94.30 ± 2.23^d^		5	100.61 ± 3.45^d^
PMSF	2.5	-3.47 ± 1.53^ g^	Mercaptoethanol	2.5	395.44 ± 11.56^b^
	5	4.81 ± 3.41^ fg^		5	444.67 ± 0.78^a^

Superscript letter(s) a, b, c, d, e, f and g down the column were used to indicate significant difference at *P* < 0.05

**Table 5:** Effect of various metal ions on the keratinase

Metal ion	Concentration (M)	Residual activity (%)	Metal ion	Concentration (M)	Residual activity (%)
None	–	100.00 ± 4.75^bc^			
Na^2+^	0.025	96.00 ± 9.64^bcd^	Si^2+^	0.025	114.76 ± 9.21^b^
	0.25	79.38 ± 5.55^cde^		0.25	116.05 ± 4.77^b^
	2.5	74.97 ± 2.05^cde^		2.5	57.48 ± 1.81^ef^
Mn^2+^	0.025	102.97 ± 22.35^bc^	Co^2+^	0.025	91.58 ± 11.57^bcd^
	0.25	260.72 ± 15.56^a^		0.25	43.89 ± 6.27^fg^
	2.5	236.62 ± 23.84^a^		2.5	40.86 ± 4.03^fg^
Mg^2+^	0.025	84.25 ± 0.64^cde^	Al^3+^	0.025	96.87 ± 1.25^bc^
	0.25	83.33 ± 1.87^cde^		0.25	11.17 ± 1.16^h^
	2.5	57.89 ± 0.98^ef^		2.5	21.53 ± 6.69^gh^
Ca^2+^	0.025	84.25 ± 5.00^cde^	Cu^2+^	0.025	− 0.37 ± 0.87^h^
	0.25	75.28 ± 12.16^cde^		0.25	7.73 ± 2.64^h^
	2.5	10.45 ± 5.13^h^		2.5	6.61 ± 2.07^h^
K^+^	0.025	83.74 ± 5.12^cde^			
	0.25	80.20 ± 0.62^cde^			
	2.5	67.43 ± 2.40^def^			

Superscript letter(s) a, b, c, d, e, f, g and h down the column were used to indicate significant difference at *P* < 0.05

### Feather degradation mechanism

**Fig. 5:**
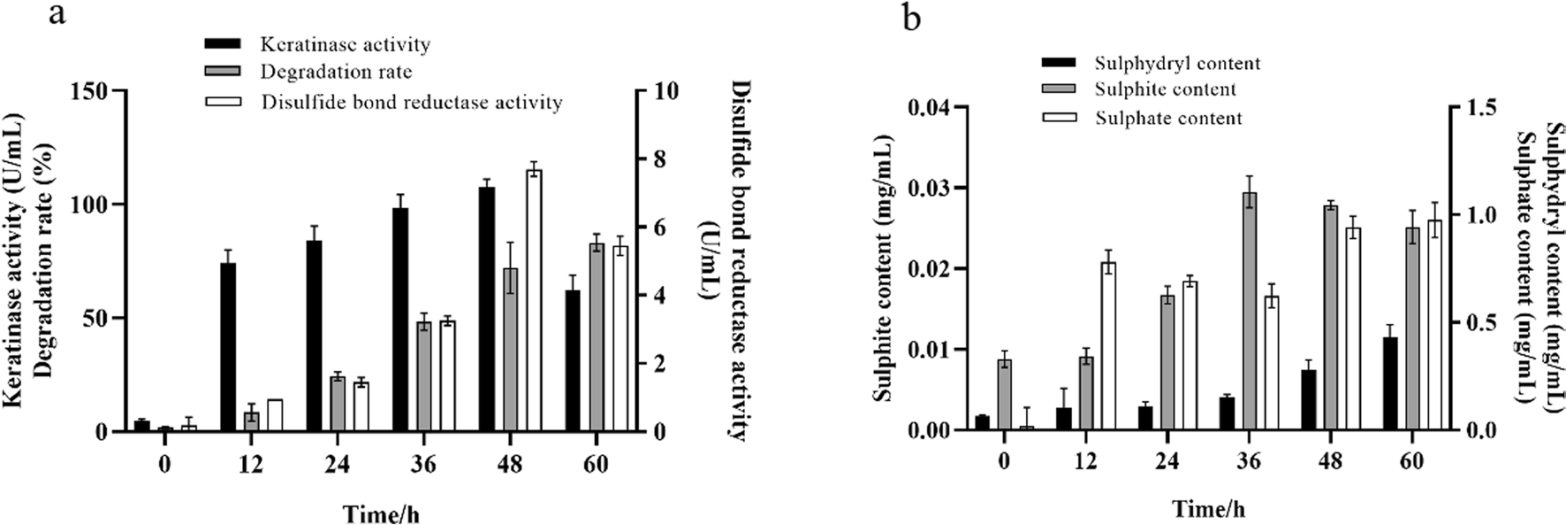
Feather degradation mechanisms of *B. tropicus* Gxun-17 during 60 h fermentation period: **a** Keratinase activity, disulphide reductase activity and feather degradation rate; **b** contents of sulphur-containing compounds

As shown in Fig. 5, the activities of keratinase and disulphide bond reductase gradually increased and reached the peak at 48 h after inoculation with *B. tropicus* Gxun-17, with the highest keratinase activity reaching 107.53 U/mL and the highest disulphide bond reductase activity reaching 7.7 U/mL. After 60 h of fermentation, the activities of both enzymes declined, but keratinase activity was higher than that of the disulphide bond reductase during the whole fermentation process. The dynamic variation of feather degradation rate was different from that of the keratinase and disulphide bond reductase. The feather degradation rate increased with fermentation time and reached the highest value at 60 h, and degradation rate was 82.23%. Except for sulphite content, the contents of sulphate and sulphydryl increased as time progressed. Sulphite was detected in the fermentation broth at 0 h, but the concentration was low. Time, the sulphite content increased slowly with the prolongation of fermentation time, and it reached the maximum at 36 h, which was 0.03 mg/mL. After 60 h of fermentation, the sulphite content decreased. However, the content of sulphate and sulphydryl reached the peak value, the highest sulphate content was 0.98 mg/mL, and the highest sulphydryl content was 0.43 mg/mL.

## Discussion

In this study, a novel *B. tropicus* Gxun-17 with high keratinase production was selected through primary screening and rescreening from the soil sample of a marine duck farm of Beibu Gulf in Guangxi, China. Currently, the research on *B. tropicus* mainly focuses on the biological activity including degradation of lignin and low-density polyethylene [23, 24]. However, the research on the degradation function of feathers has not been reported. Similar to this study, other reports revealed that feather keratin-degrading abilities in bacteria had been observed mostly in strains of *B. licheniformis*, *B. subtilis*, *B. cereus* and other *Bacillus* sp. [6–8]. Most feather-degrading bacteria studied today were isolated from terrestrial environments, such as *B*. *cereus* isolated from a poultry dump area, *Vibrio* sp. kr2 isolated from decomposing feathers at a poultry processing plant and *Pseudomonas aeruginosa* isolated from a detergent-contaminated ponds [3, 25, 26]. Few studies have explored the diversity of feather-degrading bacteria from marine environments. However, these environments are extremely complex and host a broad spectrum of species. Marine microorganisms are clearly a promising source of novel feather-degrading bacteria given their adaption to low temperature, high salinity, high pressure and oligotrophic conditions typical of the marine environment, and their enzymes are potentially very attractive for biotechnology applications owing to their stability and salt tolerance and other properties [27]. Comparison of biochemical characterisation of *B. tropicus* Gxun-17 and some microbial keratinase is presented in Table 6. The results showed that the keratinase activity (112.57 U/mL) of *B. tropicus* Gxun-17 was higher than that of some strains. Similar to most strains, the optimal temperature of the keratinase was approximately 60 °C, the optimal pH was around 7.0, and some metal ions and chemical reagents could improve the enzyme activity.

**Table 6:** Comparison of biochemical characterisation of *B. tropicus* Gxun-17 and some microbial keratinase

Microbial source	Activity (U/mL)	Optimum temperature (℃)	Optimum pH	Promoter	References
*B. tropicus* Gxun-17	112.57	60	7.0	Mn^2+^ and mercaptoethanol	This study
*B. licheniformis* ALW1	72.2	65	8.0	–	[8]
*Ochrobactrum*	117	40	9.0	K^+^, Na^+^, Ca^2+^, Mg^2+^, Dithiothreitol (DTT) and Urea	[13]
*Bacillus* sp. NKSP-7	139.35	65	7.5	Ca^2+^, Cd^2+^, Na^+^, Mn^2+^, sodium sulfite and mercaptoethanol	[19]
*B. zhangzhouensis*	117.04	60	9.5	Ca^2+^, K^+^, Na^+^, Mn^2+^ and DTT	[29]
*Thermoactinomyces* sp. RM4	–	80	10.0	Na^+^, K^+^, DTT, mercaptoethanol and toluene	[32]
*B. subtilis* P13	–	65	7.0	TritonX 100	[38]
*B. subtilis* KD-N2	–	55	8.5	SDS, EDTA, DTT, ammonium sulfamate and mercaptoethanol	[40]
*B. licheniformis*	10.76	60	7.0	Zn^2+^ and Mg^2+^	[45]

− : No mention

Orthogonal test results showed that maltose concentration, MgSO_4_ concentration, feather concentration and initial pH exerted significant effects to the keratinase with carbon source forming a major contributor. Carbon source is an important affecting factor of bacteria growth and production of metabolites. In this research, the addition of maltose significantly increased the enzyme activity and the level of enzyme-producing capacity, and the optimum maltose concentration was 10 g/L. This finding was consistent with the research results of Li et al., which indicated that maltose could provide the microorganisms with the energy needed for growth and metabolism and the carbon skeleton for synthetic products [28]. MgSO_4_, as cofactors of keratinase, also had a significant activation on the enzyme activity of *B. tropicus* Gxun-17, which might be due to the fact that Mg^2+^ could activate the active centre of the enzyme or participate directly in the composition of the active centre. This finding was consistent with the results of *Amycolatopsis* sp. strain MBRL 40 [29]. Most bacteria were reported to achieve maximum keratinase activity at feather concentrations of 5–20 g/L [30–32]. In this study, the optimum feather concentration of *B. tropicus* Gxun-17 was 15 g/L, and the enzyme activity was decreased when the concentration was 25 g/L. The reason might be that higher feather concentrations led to the relatively high viscosity of the fermentation broth, which affected the supply of oxygen in the fermentation system and thus influenced the growth of bacteria and the secretion of keratinase [33]. Moreover, neutral to weakly alkaline pH was more suitable for bacteria including *B. tropicus* Gxun-17 to produce keratinase [34–36]. Some reports have shown that the keratinase activity of *B. licheniformis* ALW1 reached 72.2 U/mL, that of *B. zhangzhou* reached 49.96 U/mL and that of *S. maltophilia* DHHJ reached 10.0 U/mL by optimising fermentation conditions and fermentation medium [8, 10, 11]. After optimisation, the keratinase activity of *B. tropicus* Gxun-17 in this study reached 112.57 U/mL, which was 3.18-fold higher than the activity of basic medium.

The results of enzymatic properties showed that the optimum pH and temperature of keratinase from *B. tropicus* Gxun-17 were 7.0 and 60 °C, respectively, which were consistent with the optimum pH and temperature of keratinase from most bacteria [13, 14, 19]. Similar to most keratinase of *Bacillus* sp., the enzyme was inhibited by PMSF [19, 37, 38]. It belonged to serine protease. Isopropanol intensively inhibited the enzyme activity; the enzyme activity was 13.91% in the presence of isopropanol at 5% concentration, which might be related to the reduction in the surrounding water content in the enzyme micro environment by the polar compound [39]. The reducing agent mercaptoethanol had an activating effect on the enzyme activity, and it was improved by 444.67% at concentration of 5%, which was significantly higher than the reported result of Cai et al. [40]. This activation action was attributed to impairing or even breaking of disulphide bonds in the keratin. Si^2+^ at concentrations of 0.025 and 0.25 M increased enzyme activity by approximately 15%. Mn^2+^ at concentrations of 0.25 and 2.5 M enhanced enzyme activity by more than 230%, which indicated that Mn^2+^ was essential for the enzyme activity and stability [11]. The enzyme was obviously inhibited by Mg^2+^, Ca^2+^, Co^2+^, K^+^ and Cu^2+^. When the concentration of Cu^2+^ was 0.025 M, the enzyme was nearly completely inhibited. Most of the heavy metal ions had an inhibitory effect on keratinase activity in most studies because the ions might have bound with catalytic residues at the enzyme active site to hinder the association of enzyme and substrates [41–43]. Studies also demonstrated that the keratinase was similar to enzyme from *Thermoanaerobacter* sp. strain 1004–09 and displayed better salt tolerance, which indicated that many keratinases from marine were adapted to the salt environment [44]. The optimal substrate for the enzyme was casein, the V_max_ of the enzyme was 0.01 mg/(mL·min) and the K_m_ was 15.24 mg/mL. The K_m_ of casein hydrolysed by keratinase from *B. licheniformis* was 0.22 mg/mL [45]. Therefore, the K_m_ varied with the catalytic ability of keratinase from different sources to casein.

To date, four theories regarding the feather degradation mechanisms are available, namely, biological membrane potential theory, mechanical pressure theory, enzymolysis theory and thiolysis theory [20]. However, the core of each theory is the fracture of disulphide bonds. In this study, *B. tropicus* Gxun-17 produced a large number of keratinase and disulphide bond reductase during the fermentation process, with the highest enzyme activities of 107.53 and 7.7 U/mL, respectively. Therefore, the two enzymes were simultaneously involved in the feather degradation process. Yamamura et al. showed that keratinase and disulphide bond reductase did not work alone but in cooperation when feather was degraded. After mixing of the two enzymes, the enzyme activity was increased by more than 50-fold [21]. With prolongation of the fermentation time, the enzyme activities decreased, whilst the feather degradation rate was continuing to increase. Thus, feather degradation mechanism could have an action other than the effects of the two enzymes. The detection of sulphur-containing compounds during the fermentation process showed that the sulphite began to rise slowly after inoculation with *B. tropicus* Gxun-17. It reached the maximum value of 0.03 mg/mL at 36 h, and it was accompanied with the increase in the contents of sulphydryl and sulphate, which was similar to the research result of Onifade et al. [22]. The breaking of disulphide bonds was related to not only enzymes but also some chemical reducing agents including sulphite, which led to protein denaturation. Therefore, the degradation of feathers by *B. tropicus* Gxun-17 might be closely related to the enzymolysis and thiolysis, and the specific degradation mechanism required further study.

## Conclusion

In this study, a novel feather-degrading *B. tropicus* Gxun-17 was obtained from marine environment. Compared with terrestrial microorganisms, the strain adapted to special environment, such as high salt, low temperature and oligotrophicity, had the advantages of high activity and strong stress resistance. The keratinase produced by the strain had a wide range of temperature and pH, and it had a certain salt tolerance, which belonged to serine protease. The enzyme-producing conditions were optimised by orthogonal test, and the keratinase activity was increased by 3.18 times, with the maximum of 112.57 U/mL. In addition, the mechanism of feather degradation by *B. tropicus* Gxun-17 was initially explored as the synergistic effect between enzymes and sulphites. This study provided a scientific basis for the utilisation of bacteria resources in the marine environment, laid the foundation for the degradation of waste feathers and the development of related feed and fertiliser products in the future and provided assistance for the biotechnology and industrial prospects of enzymes.

## Materials and methods

### Collection of soil and feather samples

Soil samples were collected from the sludge of a marine duck farm of Beibu Gulf in Guangxi, China (108.96 E, 21.63 N). The soil samples were taken from 30 cm depth from the surface of the soil in sterile polythene bags and transported to laboratory. The feathers were collected from the local poultry farm and washed thoroughly several times with water and sun dried. The treated poultry feathers were shredded into pieces approximately 2 cm long for subsequent use.

### Isolation and screening of keratinase-producing strains

For the isolation of keratinase-producing strains, 2 g of the soil sample was incubated in 18 mL of distilled water at 30 °C and 200 rpm. After 48 h, 100 μL of the sample was spread on casein agar plate (30 g casein, 1.4 g K_2_HPO_4_, 0.7 g KH_2_PO_4_, 5 g NaCl, 0.1 g MgSO_4_ and 20 g agar per litre distilled water). The hydrolysis clear zones around the bacterial colonies were observed at 30 °C for 48 h. For studying the degradation of feather, 500 μL of 12 h culture suspension of each isolate was added to 50 mL of fermentation medium (5 g NaCl, 1.4 g K_2_HPO_4_, 0.1 g MgSO_4_, 0.7 g KH_2_PO_4_ and 10 g feather per litre). The flasks were incubated at 35 °C and 200 rpm. After 48 h, the feather degradation was observed visually and keratinase activity of supernatant was measured.

### Strain identification

The selected bacterial isolate was identified by microscopic examination (Gram’s staining and scanning electron microscope), biochemical tests (urease test, oxidase test, indole test, arginine hydrolase test, hydrogen sulphide test, Voges–Proskauer test, methyl red test, gelatine test, catalase test and casein test) and 16S rRNA gene sequencing.

For 16S rRNA gene sequence analysis, the genomic DNA from the selected bacterial isolate was extracted using the bacterial genomic DNA kit. Primers 27F (5'-AGAGTTTGATCCTGGCTCAG-3') and 1492R (5'-GGTTACCTTGTTACGACTT-3') of 16S rDNA gene were used for PCR amplification. The PCR was performed in a thin-walled PCR tube containing the following: 21 μL dH2O, 25μL 2 × Taq PCR StarMix, 2 μL DNA solution, 1 μL of the 27F primer and 1 μL of the 1492R primer. The PCR conditions were initial denaturation at 95 ℃ for 4 min, followed by 30 cycles of 95 °C for 1 min, 55 °C for 1 min and 72 °C for 2 min, with a final extension step at 72 °C for 10 min. The obtained 16S rRNA gene sequences were analysed by BLAST. MEGA version 5 software was used for phylogenetic analysis following neighbour-joining method.

## Optimisation of fermentation conditions

### Single factor test

The fermentation conditions were optimised using a single factor at a time method. The feather concentration was adjusted from 5 to 25 g/L at an interval of 5 unit to establish the optimal feather concentration. Similarly, fermentation temperature (30–40 °C), initial pH (6.0–9.0) and fermentation time (12–72 h) were studied at intervals of 2.5, 0.5 and 12, respectively. The effects of additional carbon sources (glucose, sucrose, fructose, maltose, corn flour and starch) and nitrogen sources (casein, yeast extract, peptone, corn pulp, NH_4_NO_3_ and (NH_4_)_2_SO_4_) on the enzyme production were evaluated at final concentrations of 10 and 2 g/L, respectively. Next, the optimal carbon concentration (0–50 g/L) and optimal nitrogen concentration (0–10 g/L) were investigated. Inorganic salts (MnSO_4_, MgSO_4_, CuSO_4_, FeSO_4_, ZnSO_4_, AlCl_3_ and CaCl_2_) of 0.1 g/L were also added into the fermentation medium to determined, and the optimal concentration was further investigated from 0 to 0.25 g/ L with an interval of 0.5.

### Orthogonal test

An orthogonal test was performed based on the single factor test in accordance with actual conditions and cost considerations. We selected maltose concentration, feather concentration, initial pH and MgSO_4_ concentration for the 4-factor test with 3 levels of orthogonal experiments to optimise the conditions influencing the composition and improve the keratinase activity. The experiment was repeated three times, and the average was used to determine the best medium composition.

### Characterisation of keratinase

Under the optimum fermentation conditions, the supernatant was collected after the enzyme yield reached its peak, and the characteristics of keratinase were studied. The optimum pH for keratinase was investigated by incubating enzyme with casein at different pH between 6.5 and 9.0. Keratinase was preincubated at 30–90 °C prior to the activity assay, and the enzyme activity was tested at the optimum temperature. The effects of chemical reagents (EDTA, SDS and PMSF) on keratinase activity at final concentrations of 2.5 and 5 mM were also investigated, as well as the effects of reagents (mercaptoethanol, DMSO and isopropanol) at final concentrations of 2.5% and 5% (v/v). The effects of metal ions on keratinase was tested by adding various metal ions (Na^+^, Mn^2+^, Mg^2+^, Ca^2+^, K^+^, Si^2+^, Co^2+^, Al^3+^ and Cu^2+^) to the enzyme at final concentrations of 0.025, 0.25 and 2.5 M. Hair, feather, feather meal, keratin, casein and BSA were used as substrates to investigate the hydrolysis ability of keratinase. Casein at various concentrations of 0.5%, 1.0%, 1.5%, 2.0%, 2.5%, 3.0%, 3.5% and 4% (w/v) were mixed with enzyme and reacted for 10 min respectively. Then, enzyme activity was measured. K_m_ and V_max_ were calculated following the Lineweaver–Burk double reciprocal plotting method. Residual activity was measured in standard assay conditions. Appropriate controls were used.

### Feather degradation mechanism

Analysis feather fermentation broth was sampled every 12 h, for a total of six times. The keratinase activity, disulphide bond reductase activity, feather degradation rate and the contents of sulphur-containing compounds in the samples were detected.

### Determination of keratinase activity and feather degradation rate

Keratinase activity was determined using the previous methods with slight modifications [46]. Briefly, the mixture was incubated in 1.5 mL tube containing 200 μL of diluted crude enzyme and 300 μL of 2% casein substrate at 50 °C for 10 min. The enzymatic reaction was stopped by adding 500 µL of 4 mol/L trichloroacetic acid (TCA) and then centrifuged at 12,000 r/min for 10 min. Next, 1 mL of 0.5 mol/L Na_2_CO_3_ and 200 μL of folinol-phenol were added into 200 μL of supernatant successively, and they were incubated at 50 °C for 10 min. Lastly, the absorbance of the reaction solution was determined at 660 nm. The control was the inactivated enzyme solution. One keratinase unit was defined as the amount of enzyme required to hydrolyse casein to produce 1 μg tyrosine per minute at 50 °C.

To ensure the presence of thiolysis, the disulphide bond reductase activity was determined spectrophotometrically at 412 nm by measuring the yellow-coloured sulphide formed upon reduction of 5,5'-dithiobis-(2-nitrobenzoic acid) (DTNB) [47]. The mixture was incubated containing 500 μL of supernatant and 0.02 g of feather powder at 37 °C for 1 h. The enzymatic reaction was stopped by adding 4 mL of 20% TCA and then centrifuged at 12,000 r/min for 10 min. The absorbance of the mixture containing 1 mL of DTNB and 500 μL of the supernatant was measured at 412 nm. One unit of disulphide bond reductase activity was defined as an increase of 0.01 in the absorbance under described conditions.

The feather degradation rate was evaluated by weight loss method [48]. The fermentation medium was filtered through filter papers, and the feather residue was thoroughly washed with distilled water and dried and weighed to calculate weight loss. Results were expressed as percentage weight loss relative to the initial dry feather weight.

### Determination of sulphur-containing compound contents

The sulphite content was tested by pararosaniline hydrochloride method [49]. The reaction solution was mixed containing 1 mL of supernatant and 2 mL of formaldehyde–pararosaniline. The absorbance was detected at 550 nm after colour was stable. The sulphite content was calculated according to a prepared Na_2_SO_3_ standard curve.

The sulphate content was determined by barium chromate spectrophotometry [50]. Briefly, the reaction mixture was 100 μL of culture supernatant, 400 μL of H_2_O and 250 μL of BaCrO_4._ The reaction mixture was incubated at room temperature for 30 min. Next, 50 μL of calcium ammonia and 500 μL of 95% anhydrous ethanol were added to the mixture to initiate the reaction. Lastly, the reaction mixture was centrifuged at 12,000 r/min for 10 min, and the absorbance of the supernatant was determined at 420 nm. The sulphate content was calculated according to a prepared NaSO_4_ standard curve.

The release of sulphydryl into the fermentation medium was determined following the method of Ellman [47]. After the addition of 1 mL of DTNB to 500 μL of supernatant, the mixture was incubated for 10 min. The absorbance was measured at 412 nm after the development of a stable colour. The sulphydryl content was calculated according to the prepared cysteine standard curve.

### Statistical analysis of data

All experiments were performed in triplicates, and the results were taken as the mean value ± SD. For optimisation experiments, one-way ANOVA test was performed (using SPSS version 21) to calculate significant differences between means compared with control of each experiment at a 95% confidence level.

## Supplementary Information

**Additional file 1: Fig. S1.** Morphological characteristics of the isolate Gxun-17. 2. **a** Growth on the casein plate, **b** Gram’s staining image, **c** Scanning electron microscope image.

## Data Availability

The results of the datasets analyzed during the current study were included in the manuscript and the 16S rRNA sequence of *B. tropicus* Gxun-17 was submitted to the GenBank through the accession number OM256461.1. Any additional information is available from the corresponding author on reasonable request.

## Abbreviations

EDTA: Ethylene diamine tetraacetic acid
SDS: Sodium dodecyl sulfate
PMSF: Phenylmethylsulfonyl fluoride
DMSO: Dimethyl sulfoxide
TCA: Trichloroacetic acid
DTNB: 5,5′-Dithiobis-(2-nitrobenzoic acid)
DTT: Dithiothreitol
