# Supplementary material for: Isolation and identification of a feather degrading Bacillus tropicus strain Gxun-17 from marine environment and its enzyme characteristics
Source: BMC Biotechnol. 2022 Mar 20;22:11. doi: 10.1186/s12896-022-00742-w (PMC8935741; doi:10.1186/s12896-022-00742-w)
Supplement: Supplementary file 1 — Additional file 1: Fig. S1. Morphological characteristics of the isolate Gxun-17. 2. a Growth on the casein plate, b Gram’s staining image, c Scanning electron microscope image. [file 12896_2022_742_MOESM1_ESM.docx]

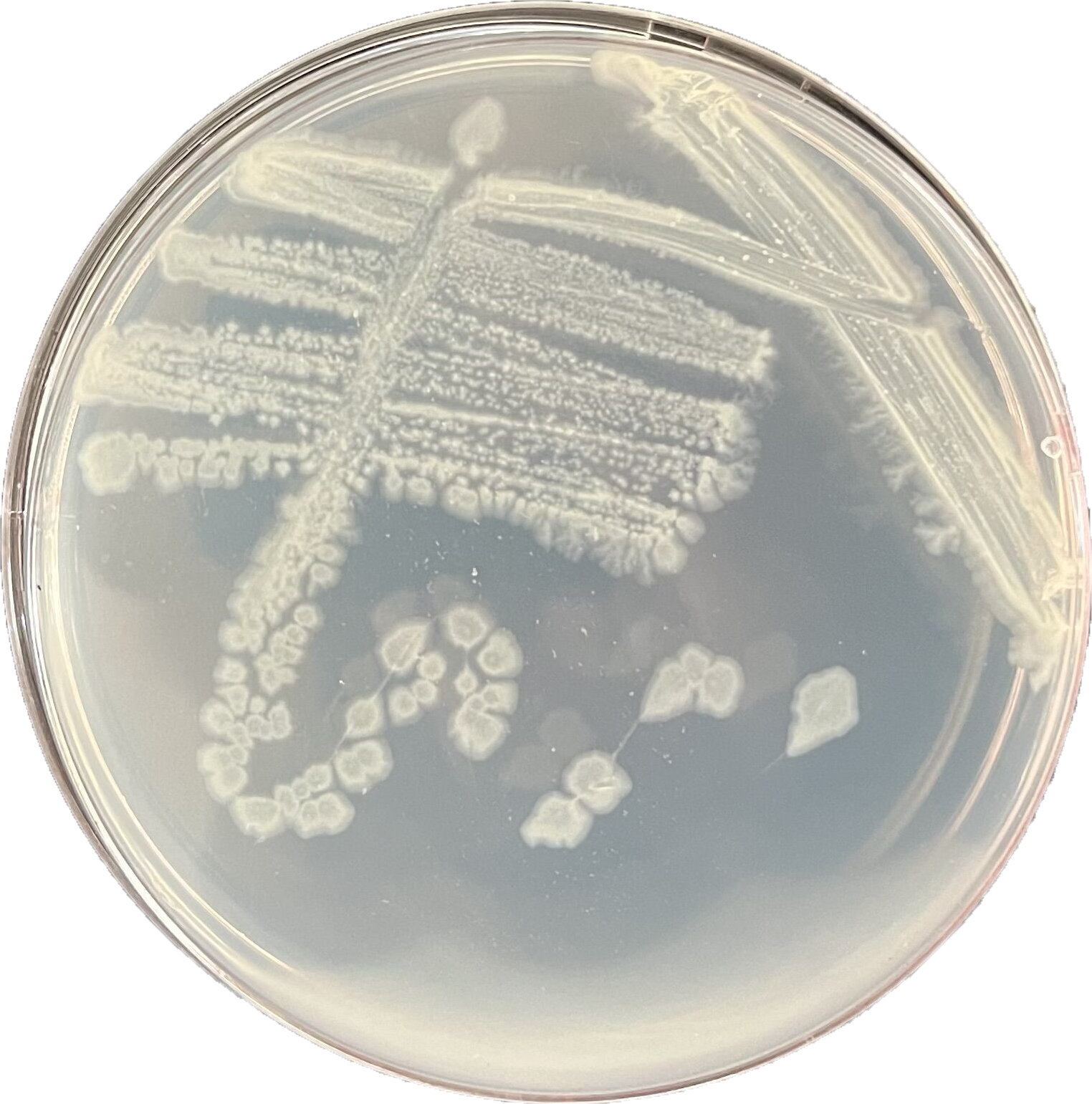


a


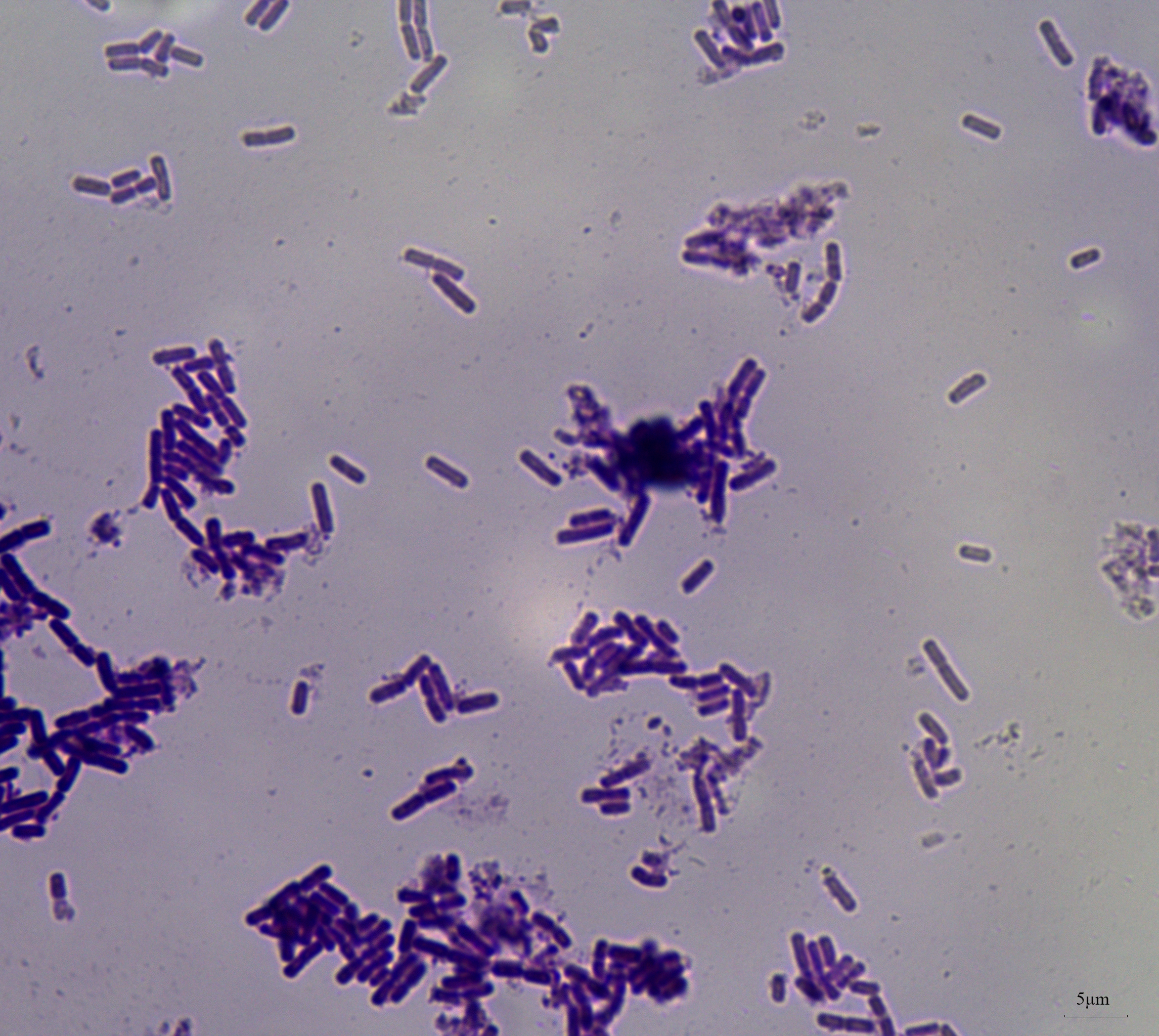


b


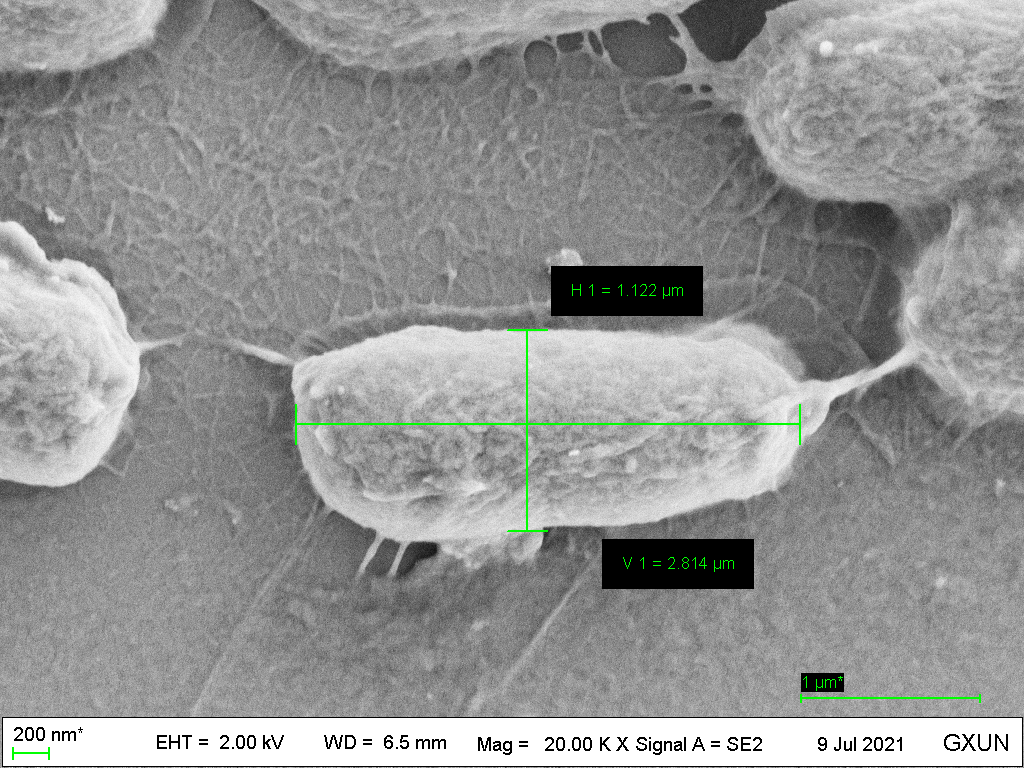


c

Fig. S1 Morphological characteristics of the isolate Gxun-17: a. Growth on the casein plate; b. Gram’s staining image; c. Scanning electron microscope image
